# Design and Early Implementation Outcomes of a Mobile Health-Enabled Social Support Intervention for Adults Living With HIV and Type 2 Diabetes in Uganda

**DOI:** 10.7759/cureus.111640

**Published:** 2026-06-28

**Authors:** Gerald Kizza, Angella Musiimenta, William Wasswa, Richard Kimera, Wilson Tumuhimbise

**Affiliations:** 1 Computer Science, Mbarara University of Science and Technology, Mbarara, UGA; 2 Research and Innovations, Angels Compassion, Mbarara, UGA; 3 Information Technology, Mbarara University of Science and Technology, Mbarara, UGA; 4 Biomedical Sciences and Engineering, Mbarara University of Science and Technology, Mbarara, UGA

**Keywords:** digital health, hiv, implementation outcomes, medication adherence, mobile health, multimorbidity, social support, treatment burden, type 2 diabetes, uganda

## Abstract

Background: Adults living with HIV and type 2 diabetes experience complex treatment demands that affect medication-taking, clinic attendance, privacy, social support, and engagement with care. Mobile health (mHealth)-enabled social support may help reduce treatment burden, but such interventions need to fit patients’ everyday realities, phone access, trusted support networks, and clinic workflow.

Objective: This study aimed to describe the user-centered design (UCD) mechanisms of an mHealth-enabled social support intervention for adults living with HIV and type 2 diabetes in Uganda and assess perceived and self-reported early implementation outcomes, including acceptability, feasibility, and appropriateness, after one month of use.

Methods: This mixed-methods formative study was conducted among 64 purposively selected participants from the HIV and diabetes clinics at Mbarara Regional Referral Hospital (MRRH), including 39 adults living with HIV and type 2 diabetes, 20 nominated social supporters, and five healthcare workers. Participants were oriented to an Mhealth-enabled social support intervention composed of short message service (SMS) reminders, educational messages, social supporter SMS prompts, discreet messaging, server-supported message routing, and clinic linkage. Patients used the intervention for one month. Post-trial interviews, focus group discussions (FGDs), brief feedback forms, observation notes, and intervention-use records were used to assess acceptability, feasibility, appropriateness, concerns, social support activation, and design refinements. Interview and focus group questions were informed by the Unified Theory of Acceptance and Use of Technology (UTAUT), and refinements followed a UCD approach. Qualitative data were analyzed thematically using NVivo, while quantitative intervention-use records and feedback responses were analyzed in Stata and summarized using frequencies, percentages, and counts.

Results: Participants perceived the intervention as useful for supporting medication-taking, clinic appointment preparation, communication with nominated social supporters, and a sense of being supported beyond the clinic. During the one-month trial, 1,092 SMS medication reminders, 74 appointment reminders, 312 educational or supportive messages, and 280 social supporter SMS prompts were delivered. Privacy-sensitive wording was strongly preferred because of the fear of unintended HIV status disclosure. The intervention was considered feasible because it used familiar channels, including SMS, voice calls, and simple app functions, although shared phone use, airtime costs, charging problems, network interruptions, language barriers, and possible clinic workload remained concerns. Participants considered the intervention appropriate because it addressed both HIV and diabetes, enabled patient-controlled social support, protected privacy, and fit existing social and clinical support practices.

Conclusion: A privacy-sensitive, low-burden intervention that combines reminders, patient-controlled supporter prompts, short educational messages, and clinic linkage could support medication-taking and care coordination for patients managing both conditions. Further implementation research is needed to refine the intervention and assess longer-term adoption, fidelity, sustainability, and preliminary impact.

## Introduction

Adults living with both HIV and type 2 diabetes face complex and continuous treatment demands. They are expected to take medicines daily, attend clinic appointments, follow dietary advice, monitor symptoms, manage transport costs, protect privacy, seek social support, and communicate with healthcare workers when problems arise [1,2]. In low-resource settings, these demands are often made more difficult by fragmented care, financial constraints, food insecurity, stigma, and limited access to continuous clinical support [2,3]. For patients managing both conditions, medication-taking is therefore not simply an individual behavior. It is part of a wider treatment workload that is managed within everyday life, often with support from family members, peers, and healthcare workers [4,5].

Burden of Treatment Theory provides a useful lens for understanding this problem because it explains how patients experience the work of managing illness in relation to the resources available to them [4,6]. Within this framework, treatment workload encompasses the practical, emotional, cognitive, social, and financial work required to manage illness and care. Correspondingly, capacity refers to the resources patients can draw upon to meet this workload, including personal ability, family support, social networks, health-system support, and material resources [4,6]. When these two elements fall out of balance, specifically when the workload of care exceeds the patient’s available capacity, patients may struggle to take medicines consistently, attend appointments, and remain engaged in care [4,6]. This imbalance, therefore, becomes a central mechanism through which treatment burden undermines continuity and quality of care among adults managing both HIV and type 2 diabetes.

Because treatment burden arises when workload exceeds available capacity, social support can play an important role in helping patients manage daily treatment work. Supporters may remind patients to take medicines, help them prepare for clinic visits, provide encouragement, assist with transport or food-related needs, and help them seek care when they are unwell [5,7]. However, social support is not always straightforward in the context of HIV and diabetes. Support may be helpful when it is trusted, respectful, and controlled by the patient, but it may also create risks of unwanted disclosure, stigma, pressure, or loss of privacy. This means that interventions designed to strengthen social support must also protect patient autonomy and confidentiality [8].

Mobile health (mHealth) interventions have increasingly been used to support medication adherence and chronic disease management through reminders, short message service (SMS) messages, voice calls, mobile applications, provider-patient communication, and digitally mediated social support [9]. However, digital health interventions may have limited value when they are designed without adequate attention to patients’ lived realities, phone access, literacy, language, privacy, cost, and social relationships [10]. In multimorbidity, digital tools must also avoid adding new tasks to patients who are already managing multiple conditions. Instead, they should reduce treatment workload and strengthen the support systems patients already use.

From a health and medical informatics perspective, this study is positioned within patient-centered informatics, multimorbidity informatics, and human-centered health technology. Patient-centered informatics emphasizes the design and use of information systems that support patients’ everyday health work, communication, engagement, and decision-making, rather than only serving institutional data needs [11]. In multimorbidity care, informatics tools must also support coordination across conditions, routines, caregivers, and clinical services, because patients often manage overlapping treatment tasks and fragmented care demands. Human-centered health technology further emphasizes that digital interventions should be designed around users’ needs, capabilities, constraints, privacy concerns, and care contexts [12].

For adults living with both HIV and type 2 diabetes, the usefulness of mHealth may therefore depend on more than simply sending reminders [13]. A reminder system may help patients remember medicines, but it may not address appointment preparation, fear of disclosure, communication with trusted supporters, language preferences, or the need for occasional contact with healthcare workers [8,13]. A more appropriate approach may be a mHealth-enabled social support intervention that combines reminders with patient-controlled supporter involvement, discreet communication, and clinic linkage.

Prior unpublished formative qualitative work conducted by our study team among adults living with HIV and type 2 diabetes in southwestern Uganda showed that patients were already using mobile phones informally to manage treatment burden and mobilize social support. Phones were used for reminders, appointment tracking, checking in with social supporters, communicating with healthcare workers, and seeking help when unwell.

However, phone use and social support were constrained by shared phone ownership, privacy concerns, airtime costs, charging problems, language barriers, low digital confidence, limited smartphone access, fear of disclosure, and household relationship dynamics. These constraints are consistent with previous work in Uganda and East Africa showing that mobile phone access is often socially shared rather than individually controlled, that phone ownership and number stability remain uneven in ways that affect public health service delivery, and that mobile technology use in public health raises important privacy, consent, ethical, legal, and sociocultural concerns [14-16]. Together, these findings suggested the need for a mHealth-enabled social support intervention that is discreet, low-cost, compatible with feature phones and smartphones, integrated across HIV and diabetes care, and linked to trusted supporters and healthcare workers.

This study, therefore, aimed to describe the design mechanisms of a mHealth-enabled social support intervention for adults living with HIV and type 2 diabetes and assess perceived and self-reported early implementation outcomes after one month of use. The specific outcomes were acceptability, feasibility, and appropriateness, with emphasis on how patient-centered informatics, multimorbidity support, and human-centered design principles shaped participants’ experiences of medication-taking support, patient-controlled social support, privacy protection, and care coordination.

## Materials and methods

Study design and setting

This study used a mixed-methods formative design to describe the design mechanisms and early implementation outcomes of an mHealth-enabled social support intervention for medication management among adults living with HIV and type 2 diabetes. The study involved a one-month trial run of the intervention, followed by qualitative and quantitative feedback on acceptability, feasibility, appropriateness, concerns, and design refinements. These outcomes were selected because they can be assessed during early-stage intervention testing before longer-term evaluation of adoption, fidelity, penetration, sustainability, and clinical impact.

This one-month period was considered sufficient to allow participants to experience medication reminders, appointment reminders, educational messages, social supporter SMS prompts, and clinic-linkage processes within routine care, while also enabling timely refinement of the intervention.

The study was conducted at Mbarara Regional Referral Hospital (MRRH) in southwestern Uganda. Participants were recruited from HIV and diabetes care clinics. The hospital provides long-term care to patients from rural, peri-urban, and urban communities. The setting was appropriate because adults living with HIV and type 2 diabetes receive continuous care from the hospital and must manage medication-taking, clinic visits, food-related challenges, transport costs, privacy concerns, and communication with healthcare workers within everyday life.

Implementation outcomes and measurement

Although this was a mixed-methods formative study involving a one-month trial run of the intervention, the assessment of early outcomes was guided by Proctor et al.’s implementation outcomes framework [17]. We focused on three outcomes that were appropriate for early-stage intervention testing: acceptability, feasibility, and appropriateness. Acceptability was defined as the extent to which patients, social supporters, and healthcare workers perceived the intervention as agreeable, satisfactory, and comfortable to use. Feasibility was defined as the extent to which the intervention could be used within participants’ everyday phone access, resource constraints, and clinic workflow. Appropriateness was defined as the perceived fit of the intervention for adults living with HIV and type 2 diabetes, including its fit with medication-taking routines, privacy needs, social support practices, and HIV and diabetes care [17].

Each outcome was assessed using both qualitative and quantitative data. Acceptability was assessed through post-trial interviews, FGDs, and feedback form items on perceived usefulness, ease of use, privacy, feeling supported, and willingness to continue using or supporting the intervention. Feasibility was assessed through qualitative accounts of practical barriers and facilitators, including phone sharing, airtime costs, charging problems, network interruptions, difficulty reading SMS, language preferences, and clinic workload, as well as intervention-use records showing messages delivered, clinic contact requests, timing changes, language changes, and supporter change or removal requests. Appropriateness was assessed through participant feedback on whether the intervention fitted the needs of people managing both HIV and type 2 diabetes, protected privacy, supported patient-controlled social support, and aligned with existing social and clinical support practices.

The closed feedback items used to summarize acceptability, feasibility, and appropriateness were study-specific formative items developed by the study team rather than validated scale measures. This approach was used because the purpose of the one-month trial was to obtain rapid, low-burden, context-specific feedback on intervention use, privacy, social supporter involvement, communication preferences, and practical feasibility in routine HIV and diabetes care. The items were therefore not intended to generate psychometric scale scores.

Reporting of the digital health intervention

This was guided by the mobile health Evidence Reporting and Assessment (mERA) checklist for mobile phone-based health interventions [18]. The checklist informed reporting of the intervention components, delivery platform, message tailoring, technology infrastructure, privacy safeguards, intervention exposure, and implementation context. This strengthened transparency and completeness in describing how the intervention was designed, delivered, and assessed.

Study participants

Between March 2026 and May 2026, we purposively recruited 64 participants, including 39 adults living with HIV and type 2 diabetes, 20 nominated social supporters, and five healthcare workers involved in HIV and/or diabetes care. Patient participants were eligible if they were (1) aged 18 years or older, (2) receiving care for both HIV and type 2 diabetes at MRRH, (3) willing to participate in a one-month intervention trial, and (4) able to use a mobile phone either directly or with support. Social supporters were trusted individuals nominated by patient participants, including spouses, adult children, relatives, friends, neighbors, or other persons who supported patients in daily treatment routines. Healthcare workers included providers involved in HIV care, diabetes care, counselling, pharmacy services, or chronic care follow-up.

Intervention technology

**Figure 1:**
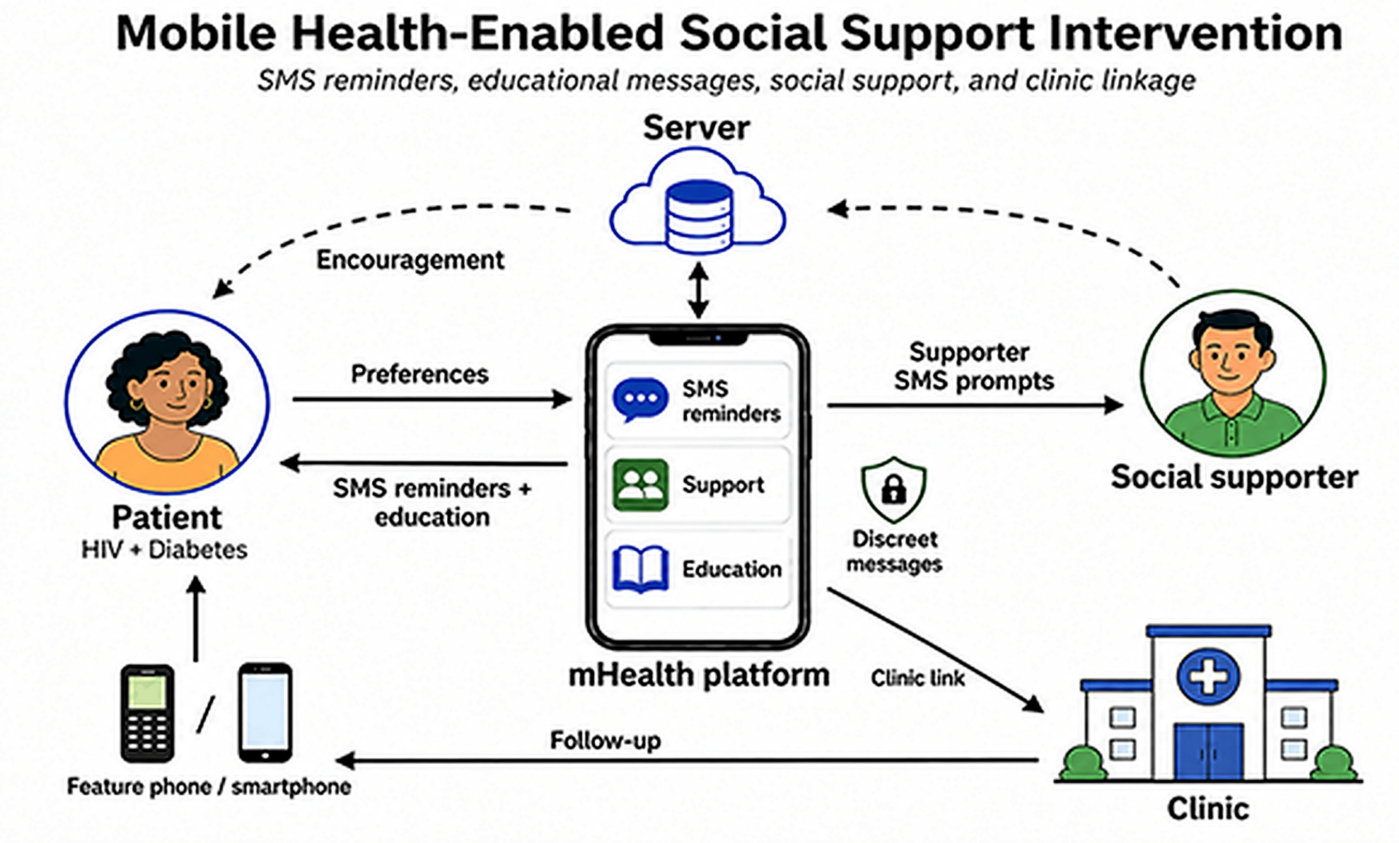
Mobile health-enabled social support intervention workflow

As shown in Figure 1, the mHealth-enabled social support intervention is composed of (1) a patient-facing mobile phone access route, (2) an mHealth platform, (3) a server, (4) SMS reminders and educational messages, (5) social supporter SMS prompts, and (6) a clinic-linkage function. The intervention is designed to support adults living with HIV and type 2 diabetes in medication-taking, appointment preparation, social support, and clinic communication using feature phones and smartphones.

The patient-facing access route was designed as an SMS-first phone-based system that could be used on both feature phones and smartphones. This design choice was intentional because participants differed in phone ownership, internet access, literacy, and digital confidence. All core intervention functions, including medication reminders, appointment reminders, short educational or supportive messages, social supporter SMS prompts, and clinic-linkage requests, were therefore delivered through SMS and did not require internet access. Feature-phone users received SMS messages and voice-call support where needed, while smartphone users received the same SMS-based functions and, where feasible, could use a simple mobile interface to view reminders, confirm selected preferences, or request support. The smartphone route was included to accommodate participants who already used smartphones and preferred app-based interaction, but it was not required for participation because the intervention was designed to remain functional through SMS alone.

The mHealth platform is the central component that manages patient preferences and coordinates message delivery. At enrolment, each patient selects preferred reminder times, language, message wording, communication channel, and whether to involve a nominated social supporter. The platform uses these preferences to deliver SMS reminders and educational messages to patients, send social supporter SMS prompts where consent has been given, and support clinic linkage when follow-up is needed.

Operationally, reminder scheduling was personalized during enrolment. Each patient was asked to indicate the usual time they took their medicines, preferred reminder time, preferred language, and whether the message should be neutral or more direct. These preferences were entered into the mHealth platform and used to create an individual reminder schedule linked to the patient’s study identification number and phone number. The server then triggered SMS reminders according to the saved schedule and routed them through the Yo! Uganda SMS gateway to the patient’s phone. Where a patient requested a timing change during the one-month trial run, the study team updated the schedule in the platform, and subsequent reminders followed the revised timing.

Digital workflow and operational procedures

During enrolment, each patient’s medication routine, preferred reminder time, language, wording, communication channel, clinic appointment date, and supporter preference were recorded and manually configured in the mHealth platform using the patient’s study identification number and phone number. Medication reminders were triggered at patient-selected fixed times, while appointment reminders were scheduled according to clinic appointment dates. Any requested changes in timing, language, or supporter involvement during the one-month trial run were updated by the study team, and subsequent messages followed the revised settings.

Supporters were linked to patients only after patient nomination and consent. The supporter’s phone number was linked to the patient’s study identification number, and supporters received general, non-diagnostic social supporter SMS prompts rather than the patient’s own messages. These prompts encouraged respectful check-ins, appointment preparation, encouragement, or practical support, while avoiding HIV status, diabetes status, medication names, clinic details, or other identifying clinical information.

Clinic-linkage alerts were generated when patients requested clarification, reported difficulty, needed follow-up, or when intervention-use records showed a reminder delivery or follow-up problem requiring study-team review. A reminder delivery or follow-up problem was defined as a scheduled reminder-related event requiring review, such as a failed or undelivered SMS delivery report, a participant report that a reminder was not received, or a clinic-related concern requiring follow-up. These alerts did not indicate confirmed missed medication doses. Alerts were reviewed by authorized study team members or clinic contact persons before follow-up. Healthcare workers responded mainly through phone calls or SMS, depending on urgency, patient preference, and network availability. Follow-up actions were documented in intervention-use records using study identification numbers.

The server stored only information needed for intervention delivery and monitoring, including patient preferences, supporter linkage status, consent status, reminder schedules, clinic appointment dates, message logs, clinic-linkage requests, and preference changes. Access was restricted to authorized study team members using password-protected accounts. Participant names and clinical identifiers were not included in outgoing SMS content, and intervention-use records were reviewed using study identification numbers to reduce exposure of identifiable health information.

The server supports the mHealth platform by storing patient preferences, managing message schedules, routing SMS reminders and educational messages, recording social supporter SMS prompts, and maintaining intervention-use records. These records include SMS reminders delivered, educational messages sent, social supporter SMS prompts, clinic-linkage requests, reminder timing changes, language changes, and supporter change or removal requests.

To protect participant confidentiality, the server was configured to store only study identification numbers, phone numbers, message preferences, reminder schedules, supporter linkage status, and intervention-use records required for message delivery and monitoring. Access to the server dashboard was restricted to authorized study team members using password-protected accounts. Participant names and clinical identifiers were not included in outgoing SMS content. Intervention-use records were reviewed using study identification numbers, and access to identifiable contact information was limited to staff responsible for enrolment, message scheduling, and follow-up.

SMS delivery is enabled through Yo! Uganda Limited, which is located in Kampala, Uganda. Yo! Uganda Limited is a technology solutions company that provides application programming interface services for sending SMS messages through major telecom providers in Uganda, including MTN and Airtel. The intervention uses this SMS gateway to route SMS reminders, educational messages, and social supporter SMS prompts from the server to patients’ and supporters’ mobile phones. This enables the intervention to reach both feature-phone and smartphone users without requiring constant internet access. SMS confidentiality safeguards were built into message design. Messages used neutral wording and avoided direct reference to HIV, diabetes, medicines, clinic attendance, or disease status unless a participant explicitly preferred more direct wording.

Message tailoring was operationalized during enrolment by asking patients to choose their preferred language, reminder timing, communication channel, and message wording. Patients were selected from predefined message options rather than writing fully customized messages. The predefined options differed mainly by privacy level. For example, a discreet message could read, “This is your reminder for today’s routine,” while a more direct message could read, “Please remember to take your medicine as agreed with the clinic.” For appointment reminders, a discreet option could read, “Please prepare for your scheduled visit,” while a more direct option could read, “Please prepare for your clinic appointment.” Patients who were concerned about phone sharing or unintended disclosure were encouraged to choose neutral wording, while those who preferred clearer reminders could choose more direct wording. Examples of the predefined SMS message options, including discreet and more direct wording variants for medication reminders, appointment reminders, educational messages, and social supporter prompts, are provided as Supplementary Files.

The SMS reminder and educational message component supports medication-taking, appointment preparation, and self-management. SMS reminders are sent to help patients remember medication-taking and prepare for clinic visits. Educational messages provide brief, locally understandable information on medication routines, food and medicine timing, clinic attendance, and living with both HIV and type 2 diabetes. Messages are designed to be discreet and avoid direct mention of HIV, diabetes, medicines, or clinic attendance unless the patient prefers explicit wording.

The social supporter SMS prompt component enables patients to nominate one trusted social supporter to receive selected prompts. These prompts guide the supporter to provide encouragement, respectful check-ins, medication-related support, appointment preparation support, or practical help. Supporter involvement is patient-controlled; patients decide whether to involve a supporter, what information the supporter receives, and whether supporter involvement should continue, change, or stop.

The clinic-linkage function enables patients to connect with healthcare workers or clinic contact persons when clarification or follow-up is needed. This function supports patients who are unwell, uncertain about medication timing, at risk of missing appointments, or in need of additional guidance. The clinic can then provide follow-up, completing the support loop between the patient, the mHealth platform, the social supporter, and the health facility.

Figure 1 shows the mHealth-enabled social support intervention workflow. The figure shows how patient preferences collected at enrolment were entered into the mHealth platform, stored on the server, converted into individualized reminder schedules, routed through the Yo! Uganda SMS gateway, and delivered to patients and nominated social supporters. It also shows how clinic-linkage requests were directed to healthcare workers or clinic contact persons for follow-up.

In practice, the workflow began with enrolment and preference setting. Patient preferences were entered into the mHealth platform, where they informed the timing, language, wording, and communication channel for messages. The server used these settings to trigger scheduled SMS medication reminders, appointment reminders, educational messages, and consent-based social supporter SMS prompts. Messages were then routed through the SMS gateway to patients and nominated supporters. When patients requested clarification, reported difficulty, or needed follow-up, the clinic-linkage function enabled the study team or clinic contact person to respond.

Design mechanisms

**Figure 2:**
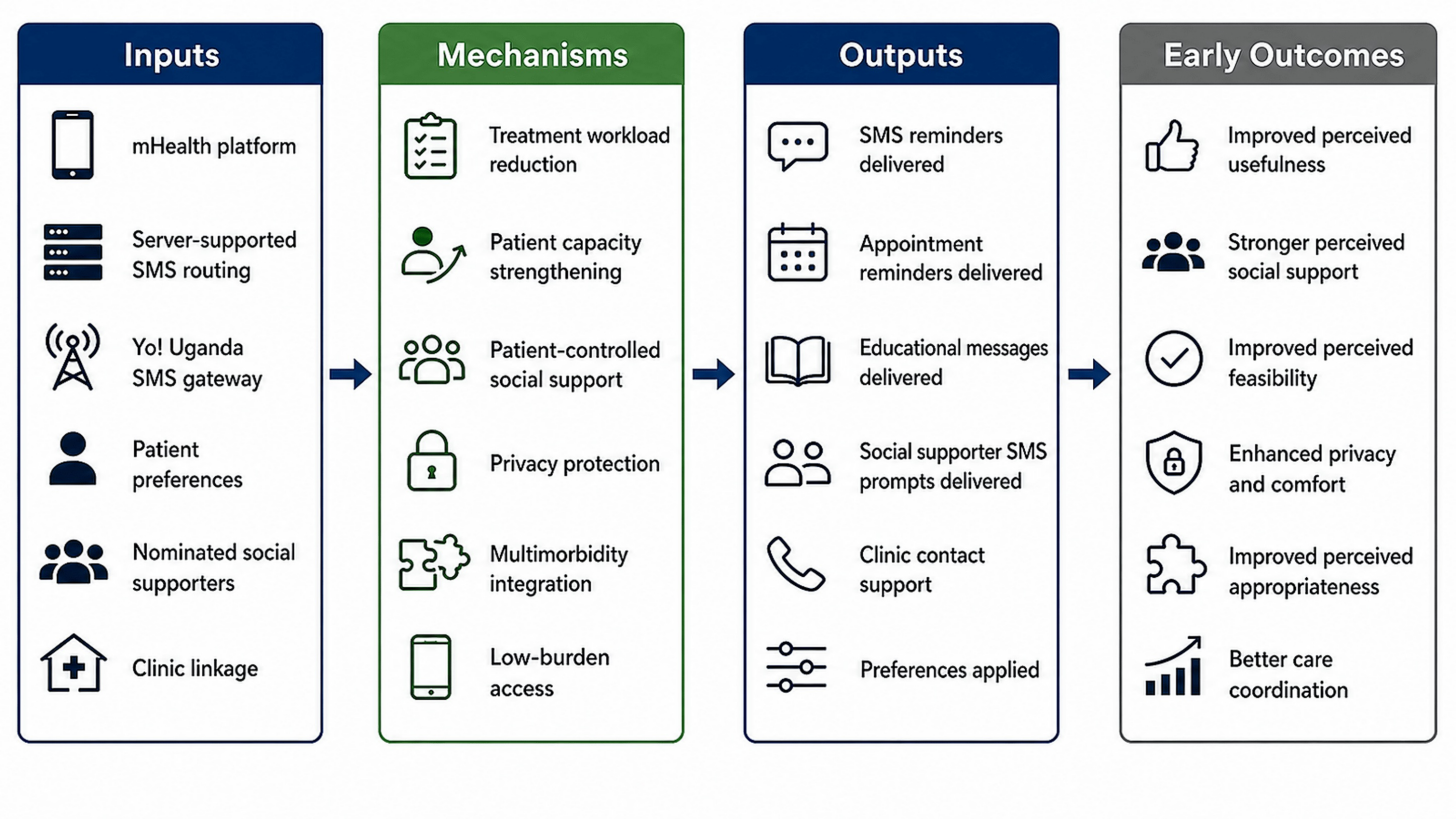
Intervention logic model for the mobile health-enabled social support intervention

The intervention was guided by six design mechanisms: (1) Treatment workload reduction focused on reducing the cognitive and organizational work involved in remembering medicines, tracking appointments, coordinating support, and seeking help. (2) Patient capacity strengthening focused on improving patients’ ability to manage treatment demands by linking them to trusted supporters and healthcare workers. (3) Patient-controlled social support activation ensured that support was activated only with the patient’s consent. Patients could decide who should support them, what information should be shared, and whether supporter involvement should continue, change, or stop. (4) Privacy protection was addressed through discreet wording, patient-selected settings, and optional supporter involvement. (5) Multimorbidity integration ensured that the intervention supported both HIV and type 2 diabetes care rather than treating the conditions as separate digital tasks. (6) Lastly, low-burden access ensured that the intervention could work through SMS, voice calls, supporter prompts, and simple smartphone functions. To clarify how the intervention was expected to work, we developed an intervention logic model linking the main intervention inputs, underlying mechanisms, outputs, and early formative outcomes (Figure 2).

As shown in Figure 2, the intervention draws on several inputs, including the mHealth platform, server-supported SMS routing, the Yo! Uganda SMS gateway, patient preferences, nominated social supporters, and clinic linkage. These inputs were intended to activate mechanisms related to treatment workload reduction, patient capacity strengthening, patient-controlled social support, privacy protection, multimorbidity integration, and low-burden access. In turn, these mechanisms were expected to generate outputs such as SMS reminders, appointment reminders, educational messages, social supporter SMS prompts, clinic contact support, and preference-based tailoring, which would contribute to early formative outcomes including improved perceived usefulness, social support, feasibility, privacy and comfort, appropriateness, and care coordination.

Study procedures

Patients were first oriented to the intervention by the study team. During enrolment, each patient selected preferred reminder times, preferred language, communication channel, message wording, and whether to involve a nominated supporter. Patients who agreed to involve a supporter identified one trusted person. Social supporters were then oriented on how to provide respectful, nonjudgmental, and privacy-sensitive support.

Supporter messaging was activated only after the patient provided clear consent and nominated the supporter. Patients were informed about the type of messages that could be sent to supporters, the purpose of supporter prompts, and the possibility of changing or stopping supporter involvement at any time. Social supporters also provided consent before receiving SMS prompts. Supporter messages were phrased in general, nondiagnostic language and did not disclose the patient’s HIV status, diabetes status, medication names, or clinic details. The orientation emphasized that supporter involvement was intended to encourage and assist the patient, not to monitor, blame, or control them.

The intervention was implemented for one month. During this period, patients received medication reminders, appointment reminders, and short supportive or educational messages. Where applicable, nominated supporters received selected prompts to encourage, remind, or check on patients in a respectful way. Healthcare workers or clinic contact persons were available to provide clarification or follow-up when patients requested support or when clinic-related concerns arose.

Data collection and user-centered intervention development

We used posttrial interviews and FGDs to solicit participants’ perceptions about the initial version of the mHealth-enabled social support intervention. The development of questions for the interviews and focus group discussions was informed by the UTAUT model, with specific focus on participants’ perceived usefulness of the intervention, ease of use, facilitating conditions, social influence, and anticipated challenges in using the intervention [19]. The questions also focused on early implementation outcomes, including acceptability, feasibility, and appropriateness as defined by Proctor [17].

Subsequent refinements of the intervention were developed using a user-centered design (UCD). UCD is an iterative intervention development process in which users’ needs, preferences, concerns, and feedback are continuously solicited and incorporated into the design process so that the intervention can work within the intended context [20,21]. With this approach, the intervention was designed and refined around users’ needs, rather than expecting patients, social supporters, and healthcare workers to adjust their routines to fit the technology.

Specifically, participants were oriented to the intervention and taken through its main functions, including receiving SMS reminders, receiving educational messages, selecting preferred message timing and language, activating or declining social supporter involvement, receiving social supporter SMS prompts, using discreet message wording, and linking with the clinic when follow-up was needed. Participants then provided feedback on how they felt about using the intervention, including its anticipated benefits and barriers; use of feature phones, smartphones, SMS, and voice calls; preferred content and timing of SMS messages; privacy and confidentiality concerns; appropriateness of involving social supporters; and the fit of the intervention within HIV and diabetes care.

The FGDs explored group-level perceptions of the intervention, including acceptability, feasibility, appropriateness, concerns, and design refinements. Post-trial interviews allowed more detailed exploration of individual experiences, especially around privacy, phone sharing, medication-taking routines, supporter involvement, and clinic linkage. Feedback from patients, social supporters, and healthcare workers was used to identify refinements needed to make the intervention more discreet, acceptable, low-burden, locally understandable, and suitable for adults living with HIV and type 2 diabetes.

Interviews and FGDs were conducted by the lead author, with support from trained research assistants fluent in Runyankole/Rukiga and English. The lead author is a PhD researcher in computing and health informatics and was not part of the participants’ routine clinical care team. Before each interview or FGD, participants were informed about the researcher’s role, the purpose of the study, confidentiality, and voluntary participation. Interviews and FGDs were conducted in the language preferred by participants, mainly Runyankole/Rukiga or English. Discussions and interviews were digitally recorded, transcribed, and translated into English where necessary. Reflexive notes were maintained during data collection and analysis to document emerging impressions, researcher assumptions, contextual issues, participant interaction dynamics, and decisions made during coding and theme development. These notes helped the study team consider how the researcher’s background in computing and health informatics, prior involvement in intervention development, and engagement with the study setting could shape the interpretation of participants’ feedback.

We also administered brief feedback forms composed of closed and open-ended questions. Information elicited by the feedback forms included participants’ phone access, preferred communication channels, preferred language, perceived usefulness of SMS reminders and educational messages, acceptability of social supporter SMS prompts, privacy concerns, feasibility challenges, and suggested intervention refinements.

Intervention-use records were also reviewed to describe participants’ interaction with the intervention. These records captured SMS reminders delivered, appointment reminders delivered, short educational or supportive messages delivered, social supporter SMS prompts delivered, clinic contact requests, Reminder delivery/follow-up problem alerts, reminder timing changes, language changes, and supporter change or removal requests.

Data analysis

We used thematic analysis to derive categories describing participants’ perceptions and experiences of the mHealth-enabled social support intervention [22]. Initially, the lead author reviewed the interview and focus group discussion transcripts, observation notes, and open-ended feedback responses for content relevant to perceived usefulness, feasibility, appropriateness, privacy concerns, social support activation, anticipated barriers, and design refinements. A codebook was then developed from the emerging concepts using an iterative process that involved developing codes, writing operational definitions, and identifying illustrative quotations.

Following completion of the codebook, transcripts and open-ended responses were coded using NVivo qualitative data analysis software version 11(Released 2015; QSR International, Burlington, MA, USA). Codes were grouped into broader categories describing perceived usefulness, feasibility, appropriateness, privacy and discretion, human and social support, and suggested intervention refinements. Interpretation was also informed by Burden of Treatment Theory by examining how the intervention reduced treatment workload, strengthened patient capacity through social support, and improved the fit between treatment demands and available resources. Quantitative data from intervention-use records and brief feedback forms were analyzed using Stata version 13 (Released 2013; StataCorp, College Station, TX) and summarized descriptively using frequencies, percentages, and counts.

To strengthen analytic transparency, the lead author maintained an audit trail consisting of the codebook, coding notes, reflexive memos, category development notes, and summaries of theme refinement decisions. Although formal intercoder agreement was not calculated, emerging codes, the codebook, and candidate themes were discussed with members of the research team to check consistency, plausibility, and fit with the data. Thematic findings were then compared with quantitative feedback items and intervention-use records to identify areas of convergence, divergence, and needed design refinement.

Qualitative reporting was guided by the Consolidated Criteria for Reporting Qualitative Research (COREQ), which supports transparent reporting of interviews and focus group discussions. The checklist informed reporting of participant selection, data collection procedures, researcher role, transcription, coding, theme development, and use of illustrative quotations [23].

Ethical considerations

Ethical approval was obtained from the Mbarara University Research Ethics Committee (REC No: MUST-2025-611), and regulatory clearance was obtained from the Uganda National Council for Science and Technology (Registration No: SIR624ES). Administrative permission was obtained from MRRH before data collection. All participants provided informed consent before participating in the study. Participants were informed about the purpose of the study, study procedures, possible risks and benefits, confidentiality, voluntary participation, and their right to withdraw at any time without affecting their care.

Given the sensitivity of HIV status, the involvement of social supporters, and the possibility of unintended disclosure through phone communication, privacy was emphasized throughout intervention design, implementation, data collection, and reporting. SMS messages used discreet wording and avoided direct mention of HIV, diabetes, medication names, or clinic attendance unless preferred by the participant. Supporter messaging was based on explicit patient nomination and consent, and supporters received only general prompts intended to encourage respectful support. Server access was restricted to authorized study team members, and intervention-use records were reviewed using study identification numbers to minimize exposure of identifiable health information.

## Results

Participant characteristics and phone access

A total of 64 participants were enrolled in the one-month intervention trial and feedback process. These included 39 adults living with HIV and type 2 diabetes, 20 nominated social supporters, and five healthcare workers involved in HIV and/or diabetes care. Overall, 61 participants completed the one-month trial and feedback activities, including 37 patients, 19 social supporters, and all five healthcare workers. Three participants did not complete the one-month follow-up activities: two patient participants and one social supporter. Reasons for noncompletion were not fully documented, although no participant reported withdrawal due to intervention-related harm. The participant groups, completion numbers, and main roles are summarized in Table 1. Phone access and communication preference data in Table 2 were collected at enrolment and are therefore reported among all 39 enrolled patients. Patient-reported acceptability, feasibility, and appropriateness items in Tables 3-4 were collected at follow-up and are therefore reported among the 37 patients who completed the one-month trial and feedback activities. Social supporter feedback in Table 5 is reported among the 19 social supporters who completed follow-up, while healthcare worker feedback in Table 6 is reported among all five healthcare workers.

**Table 1:** Participant profile

Participant group	Number enrolled	Number completing one-month trial	Main role
Adults living with HIV and Type 2 diabetes	39	37	Used the phone-based medication support intervention
Nominated social supporters	20	19	Received selected prompts and supported patients
Healthcare workers	5	5	Reviewed clinic fit, feasibility, and workflow
Total	64	61	

**Table 2:** Patient phone access and communication preferences

Characteristic	Number, n (%)
Patients enrolled	39 (100.0)
Used feature phones	21 (53.8)
Used smartphones	18 (46.2)
Preferred SMS reminders	24 (61.5)
Preferred voice-call reminders	9 (23.1)
Preferred app notifications	6 (15.4)
Preferred Runyankole/Rukiga messages	31 (79.5)
Preferred English messages	8 (20.5)
Preferred discreet/neutral wording	35 (89.7)
Chose to involve a social supporter	20 (51.3)
Did not involve a social supporter	19 (48.7)

**Table 3:** Patient-reported acceptability, feasibility, and appropriateness items

Outcome item	Agree/strongly agree, n (%)
The reminders helped me remember my medicines	34/37 (91.9)
The intervention helped me prepare for clinic appointments	31/37 (83.8)
The messages were easy to understand	32/37 (86.5)
The timing of reminders fitted my daily routine	29/37 (78.4)
The wording protected my privacy	35/37 (94.6)
The intervention was simple to use	30/37 (81.1)
The intervention helped me feel supported	28/37 (75.7)
I would continue using the intervention if available	33/37 (89.2)

**Table 4:** Reported feasibility challenges among patients completing the trial

Feasibility challenge	Patients reporting challenge, n (%)
Shared phone use	12/37 (32.4)
Charging problems	14/37 (37.8)
Airtime or data cost concerns	16/37 (43.2)
Network interruptions	10/37 (27.0)
Difficulty reading SMS	7/37 (18.9)
Fear of unintended disclosure	9/37 (24.3)
Difficulty using app features	6/37 (16.2)

**Table 5:** Social supporter feedback

Outcome item	Agree/strongly agree, n (%)
The prompts helped me support the patient	17/19 (89.5)
The prompts reminded me to check on the patient respectfully	16/19 (84.2)
The intervention did not make me feel like I was controlling the patient	15/19 (78.9)
The message wording protected the patient’s privacy	18/19 (94.7)
I would continue supporting the patient through this system	17/19 (89.5)

**Table 6:** Healthcare worker feedback

Outcome item	Agree/strongly agree, n (%)
The intervention fits HIV and diabetes care needs	5
The intervention can support medication-taking outside the clinic	5
The intervention is feasible if routine reminders are automated	5
The intervention may increase workload if not well integrated	4
The intervention should generate simple follow-up lists	5
The intervention is appropriate for patients managing both conditions	5

Among the 39 patient participants enrolled, 21 (53.8%) used feature phones and 18 (46.2%) used smartphones. Most patients preferred SMS reminders (24/39, 61.5%), followed by voice-call reminders (9/39, 23.1%) and app notifications (6/39, 15.4%). Most patients preferred messages in Runyankole/Rukiga (31/39, 79.5%), while eight (20.5%) preferred English. Discreet or neutral message wording was preferred by 35 (89.7%) patients. Twenty patients (51.3%) chose to involve a nominated social supporter, while 19 (48.7%) preferred not to involve a supporter. Patient phone access and communication preferences are presented in Table 2. These findings therefore showed that the intervention needed to support both feature phones and smartphones, allow language and message preferences, and keep social supporter involvement optional and patient-controlled.

Intervention exposure during the one-month trial

During the one-month trial, the intervention delivered SMS medication reminders, appointment reminders, short educational or supportive messages, social supporter SMS prompts, and clinic-linkage support. A total of 1,092 SMS medication reminders, 74 appointment reminders, 312 short educational or supportive messages, and 280 social supporter SMS prompts were delivered. Eighteen patient-initiated clinic contact requests were recorded. These requests were mainly related to medication timing, appointment clarification, symptoms, or difficulty attending the clinic. The intervention exposure records are summarized in Table 7.

**Table 7:** Intervention exposure over one month

Intervention component	Number delivered/recorded
SMS medication reminders delivered	1,092
Appointment reminders delivered	74
Short educational/supportive messages delivered	312
Social supporter SMS prompts delivered	280
Patient-initiated clinic contact requests	18
Missed reminder follow-up alerts	26
Reminder timing changes requested	11
Language changes requested	7
Supporter change/removal requests	2

Participants also requested adjustments during the intervention period. Eleven patients requested changes in reminder timing, seven requested language adjustments, and two requested changes in supporter involvement. These changes showed the importance of allowing patients to adjust message timing, language, and social support arrangements during implementation.

Acceptability and perceived usefulness of the intervention

Acceptability was reflected in participants’ reports that the intervention was helpful, easy to understand, simple to use, supportive, and worth continuing. Among patients who completed the one-month trial run, 34 of 37 (91.9%) reported that the reminders helped them remember their medicines, while 33 of 37 (89.2%) reported that they would continue using the intervention if it were available. Thirty-one of 37 patients (83.8%) reported that the intervention helped them prepare for clinic appointments. Patient-reported acceptability, feasibility, and appropriateness items are shown in Table 3. Patients described SMS reminders as helpful in maintaining daily routines, especially when they were busy, travelling, emotionally tired, or uncertain about medication timing. Appointment reminders were also valued because they helped patients prepare for clinic visits and reduced the risk of missing refills or reviews.

One patient explained, “The reminder helped me because sometimes I am busy and time passes without noticing. When the message comes, I remember that it is time for my routine.”

Another patient described the value of appointment reminders: “The appointment reminder helped me prepare early because sometimes transport money is not easy to get on the same day.”

Social supporters also perceived the intervention as useful. Seventeen of 19 social supporters (89.5%) reported that the prompts helped them support the patient, and 16 of 19 (84.2%) reported that the prompts reminded them to check on the patient respectfully. Social supporter feedback is summarized in Table 5. Supporter prompts helped them provide encouragement and check-ins without repeatedly asking direct questions that could be perceived as intrusive or embarrassing.

One social supporter stated, “As a supporter, it helped me to check on her without sounding like I am disturbing her all the time.”

Healthcare workers viewed the intervention as useful because it reinforced counselling messages outside the clinic and created a possible pathway for identifying patients who needed additional follow-up. All five healthcare workers agreed that the intervention could support medication-taking outside the clinic, while four noted that it could increase workload if not well integrated into routine care, as shown in Table 6.

Feasibility concerns and design considerations

The intervention was considered feasible because it used familiar communication channels, including SMS, voice calls, social supporter SMS prompts, and simple app functions. Feature-phone compatibility was particularly important because many patients did not own smartphones or could not rely on internet-based communication. Participants also valued short messages, local-language communication, and flexible reminder timing.

However, feasibility was shaped by practical constraints. Sixteen of 37 patients (43.2%) reported airtime or data cost concerns, 14 (37.8%) reported charging problems, 12 (32.4%) reported shared phone use, and 10 (27.0%) reported network interruptions. Seven patients (18.9%) reported difficulty reading SMS, while six (16.2%) reported difficulty using app features. Nine patients (24.3%) reported fear of unintended disclosure. These feasibility challenges are presented in Table 4.

A healthcare worker noted, “The idea is good, but it should remain simple. If it requires internet all the time, many patients will fail to use it.”

Healthcare workers emphasized that the intervention would be feasible in routine care only if it did not create heavy additional workload. They preferred a model in which routine SMS reminders and social supporter SMS prompts were automated, while human follow-up was reserved for patients who missed appointments, failed to respond repeatedly, or requested clinical clarification.

Another healthcare worker explained: “The system can help, but it should not add too much work to the clinic. Automated reminders can continue, then health workers can follow up those who really need help.”

Participants highlighted several acceptability, feasibility, and appropriateness issues that informed minor refinements to the intervention. These issues included message length, language preference, reminder timing, voice-call options, feature-phone and smartphone access, clinic contact needs, message frequency, privacy-sensitive wording, and patient-controlled social support. The issues and corresponding refinements are summarized in Table 8. Most refinements involved adjusting message wording, timing, language, and support preferences within the existing platform. They did not require offloading and reloading the intervention on participants’ phones. The main qualitative themes, subthemes, representative quotations, and interpretations are summarized in Tables 9-10.

**Table 8:** Early implementation issues and design refinements

Issue highlighted	Refinement/action
Some patients preferred shorter and simpler messages	Keep SMS messages brief, clear, and easy to understand.
Some participants preferred local-language communication	Provide Runyankole/Rukiga message options alongside English.
Patients preferred discreet wording to reduce disclosure risk	Use neutral wording unless a patient prefers more direct wording.
Airtime or data cost concerns	Maintain SMS as the core low-cost delivery channel.
Charging problems	Avoid app-only functions and keep SMS reminders available.
Shared phone use	Use discreet messages and patient-selected privacy preferences.
Network interruptions	Use SMS-based delivery that can be received when network becomes available.
Difficulty reading SMS	Keep messages short and offer voice-call support where needed.
Difficulty using app features	Ensure all core functions remain accessible through SMS.
Concern about too many messages	Avoid excessive messaging and allow temporary pausing of reminders.
Healthcare worker concern about workload	Automate routine reminders and reserve follow-up for patients needing support.
Patients wanted support but did not want to lose control over shared information	Keep social supporter involvement optional, consent-based, and patient-controlled.
Need for clinic contact when clarification or follow-up was needed	Maintain clinic-linkage function for patient requests and targeted follow-up.
Core intervention functions were acceptable and usable during the trial run	Retain the core intervention design; only minor refinements were needed within the existing platform.

**Table 9:** Summary of qualitative themes and related intervention refinements

Qualitative theme	Meaning	Related intervention refinement
Perceived usefulness of reminders	Participants felt that reminders supported medication-taking and appointment preparation.	Retain SMS medication reminders and appointment reminders.
Privacy and discretion	Participants preferred wording that did not reveal HIV status, diabetes status, medicine use, or clinic attendance.	Use neutral wording unless a participant prefers more direct wording.
Patient-controlled social support	Supporter involvement was acceptable when patients chose the supporter and controlled what information was shared.	Keep supporter involvement optional, consent-based, flexible, and patient-controlled.
Feasibility in everyday life	Use was shaped by phone sharing, charging problems, airtime costs, network interruptions, literacy, language, and phone type.	Maintain SMS-first delivery, local-language options, and voice-call support where needed.
Clinic linkage and workload	Clinic linkage was valued, but healthcare workers preferred a system that did not increase routine workload.	Automate routine reminders and reserve human follow-up for patients needing support.

**Table 10:** Qualitative themes, subthemes, and representative quotations

Theme	Subtheme	Representative quotation	Interpretation
Perceived usefulness of reminders	Medication-taking support	“The reminder helped me because sometimes I am busy and time passes without noticing. When the message comes, I remember that it is time for my routine.”	Participants perceived reminders as useful for supporting daily medication routines.
Perceived usefulness of reminders	Clinic appointment preparation	“The appointment reminder helped me prepare early because sometimes transport money is not easy to get on the same day.”	Appointment reminders were perceived as useful for planning clinic attendance and transport.
Patient-controlled social support	Respectful supporter involvement	“As a supporter, it helped me to check on her without sounding like I am disturbing her all the time.”	Supporter prompts were perceived as helpful when they encouraged respectful check-ins.
Privacy and discretion	Avoiding unintended disclosure	“I liked that the message was not mentioning HIV. Even if someone sees it, they cannot know exactly what it is about.”	Discreet wording was important for reducing fear of unintended disclosure.
Feasibility and access	Need for simple, low-burden technology	“The idea is good, but it should remain simple. If it requires internet all the time, many patients will fail to use it.”	Participants and healthcare workers emphasized SMS-first delivery and avoidance of app-only functions.
Clinic linkage and workload	Targeted follow-up rather than routine workload	“The system can help, but it should not add too much work to the clinic. Automated reminders can continue, then health workers can follow up those who really need help.”	Healthcare workers supported automation for routine reminders and targeted human follow-up for patients needing additional support.
Patient control over social support	Support without loss of autonomy	“The message guided me to support my patient, but the patient remained the one in control.”	The supporter model was acceptable when patients retained control over supporter involvement and shared information.

Appropriateness and patient-controlled social support

Participants considered the intervention appropriate when it addressed HIV and diabetes together, protected privacy, and allowed support from trusted people without removing patient control. Patients described the integrated reminder, education, social support, and clinic-linkage functions as appropriate because they experienced HIV and diabetes medication management as part of one daily routine. Thirty-five of 37 patients (94.6%) reported that the message wording protected their privacy. Discreet wording was valued because it reduced concerns about unintended disclosure, especially where phones were shared or handled by family members.

One patient stated: “I liked that the message was not mentioning HIV. Even if someone sees it, they cannot know exactly what it is about.”

The supporter component was acceptable when patients remained in control of who was involved and what information was shared. Of the 39 patient participants, 20 chose to involve a nominated social supporter, and 280 social supporter SMS prompts were delivered during the one-month trial run. Two patients requested changes in supporter involvement, reinforcing the need to keep supporter participation flexible and patient-directed. Although the supporter component was central to the intervention design, only 20 of 39 patients chose to involve a nominated social supporter. This finding suggests that digitally mediated social support may be useful for some patients but should not be treated as universally acceptable or required. For patients who did not involve a supporter, possible concerns may have included privacy, disclosure risk, household relationship dynamics, or preference for managing treatment independently. Therefore, the intervention model should remain modular, allowing patients to use reminders, educational messages, and clinic linkage without necessarily activating supporter prompts.

Social supporters also felt that the system was appropriate because it guided them to provide encouragement and reminders without becoming harsh, intrusive, or controlling. This patient-controlled approach made social support acceptable because it strengthened help without increasing disclosure risks.

One social supporter explained: “The message guided me to support my patient, but the patient remained the one in control.”

Participants valued the intervention when it helped them remember medicines, prepare for clinic visits, receive support from trusted people, and maintain privacy without adding pressure on the clinic. Participants preferred the intervention as a human-supported and socially supportive digital tool, not as a fully automated reminder system.

## Discussion

Principal results

This formative mixed-methods study found that a mHealth-enabled social support intervention composed of SMS reminders, educational messages, social supporter SMS prompts, discreet communication, and clinic linkage was perceived as acceptable, feasible, and appropriate for supporting medication-taking and care coordination among adults living with HIV and Type 2 diabetes in Uganda. Participants reported that the intervention helped them remember medicines, prepare for clinic appointments, feel supported by trusted people, and access clarification or follow-up when needed. Intervention-use records showed that SMS medication reminders, appointment reminders, educational or supportive messages, social supporter SMS prompts, clinic contact requests, and preference adjustments were delivered during the one-month trial run. Participants also identified concerns related to unintended HIV status disclosure, shared phone use, airtime costs, charging problems, network interruptions, difficulty reading SMS, limited smartphone access, and possible clinic workload.

These findings suggest that participants perceived the intervention as more than a reminder system; they experienced it as a low-burden support pathway linking patients, trusted social supporters, and clinic follow-up. Interpreted through Burden of Treatment Theory, the reminders and appointment messages were perceived as helping participants organize medication-taking and clinic preparation, while patient-controlled supporter prompts were perceived as strengthening available support for daily treatment work. However, this formative study did not objectively measure treatment burden reduction, medication adherence, or clinical outcomes. The findings therefore support conclusions about perceived usefulness, acceptability, feasibility, and appropriateness, rather than demonstrated behavioral or clinical benefit. Privacy protection, flexible communication options, local-language messaging, and compatibility with feature phones and smartphones emerged as central design conditions for making digital support acceptable and usable in this multimorbidity context.

Comparison with prior work

Participants valued the SMS reminders, educational messages, and clinic linkage because they made medication-taking and appointment preparation feel more supported beyond the clinic. Similar work in rural Uganda has shown that SMS reminders and real-time adherence monitoring can support antiretroviral therapy adherence by providing reminders, accountability, and a sense of being connected to care [7, 24]. Mobile phone-based HIV adherence support tools in Uganda have also been reported as acceptable when they are simple, responsive to users’ needs, and able to support communication between patients and healthcare workers [13, 25].

Participants did not perceive the intervention as a reminder system alone. They valued it more when reminders were combined with educational messages, supporter involvement, and clinic linkage. This finding agrees with reviews of mHealth interventions for chronic disease management showing that digital interventions are more useful when they include interactive support, tailored communication, user engagement features, and support for self-management rather than relying only on one-way reminders [26, 27].

This reinforces the value of human-centered health technology, where intervention usefulness depends on fit with users’ routines, literacy, privacy needs, phone access, and social relationships.

Participants also valued the social supporter SMS prompts because they helped activate support from trusted people without making the support feel forced or intrusive. This finding is consistent with evidence showing that social support is important in helping patients manage treatment burden and chronic illness self-care [4]. It also aligns with broader evidence that peer and lay support can improve chronic disease self-management when support is acceptable, practical, and integrated into patients’ everyday lives [28]. In the present study, the supporter component was especially important because patients retained control over who was involved and what information was shared.

The present study adds to this work by focusing on adults living with both HIV and Type 2 diabetes. Managing more than one chronic condition often requires patients to coordinate medicines, clinic visits, symptoms, lifestyle advice, and support from other people. Reviews on digital support for multimorbidity have similarly emphasized that digital applications should support self-management while accounting for treatment burden, patient capacity, digital access, and the complexity of managing multiple conditions [29].

Privacy and disclosure concerns were central to how participants judged the intervention. Similar concerns have been reported in HIV-related mHealth studies in Uganda, where mobile phone reminders and electronic adherence technologies were acceptable but also raised concerns about confidentiality and unintended disclosure [7, 8]. Notably, digital adherence technologies may also support intentional disclosure where patients choose to involve trusted people for support [8]. In the present study, this made discreet wording and patient-controlled supporter involvement important design features.

The feasibility concerns reported in this study also reflect realities described in prior digital health work in Uganda. Participants preferred an intervention that could work through familiar channels such as SMS, voice calls, and simple app functions because phone sharing, airtime costs, charging problems, network interruptions, and limited smartphone access affected use. Similar implementation work has shown that mHealth interventions in Uganda need to consider sustainability, cost, phone access, and routine clinic workflow if they are to be maintained beyond research settings [13, 25].

Healthcare workers preferred automation for routine reminders and social supporter SMS prompts, with human follow-up reserved for patients needing clarification or additional support. This finding is consistent with routine HIV care implementation work in Uganda showing that digital adherence systems can be promising when they generate actionable information for follow-up without creating unnecessary workload for health workers [13]. Sustainable implementation may therefore depend on integrating automated support with targeted human follow-up within existing clinic workflows.

In summary, this evidence supports a model in which routine digital support is automated, social support remains patient-directed, and clinic follow-up is reserved for patients who need additional help. Such an approach may be more suitable for low-resource settings where phone access, privacy, and clinic workload shape how digital health interventions are used

Strengths and limitations

A strength of this study is that it involved patients, nominated social supporters, and healthcare workers, allowing the intervention to be assessed from multiple perspectives. This multi-stakeholder approach provided insight into patient experience, supporter involvement, and clinic workflow. Another strength is that the intervention was developed and tested within the same care context, using findings from prior formative work among adults living with HIV and Type 2 diabetes. The privacy-sensitive design, including discreet message wording and patient-controlled supporter involvement, is also an important strength because it reflects the realities of HIV-related disclosure concerns, shared phone use, and the need to protect patient autonomy.

This study also had limitations. The one-month trial period did not allow assessment of longer-term implementation outcomes such as adoption, fidelity, sustainability, or clinical impact. The study focused on early perceived and self-reported implementation outcomes rather than objective measures of medication adherence, glycemic control, or HIV clinical outcomes. Because participants were oriented to the intervention, supported during the one-month trial, and then asked to provide feedback to the study team, the acceptability, feasibility, and appropriateness findings may have been influenced by social desirability or acquiescence bias. Therefore, the high agreement rates should be interpreted as formative perceptions rather than definitive evidence of intervention effectiveness.

The qualitative analysis was led by a single primary coder, which may have limited analytic reproducibility. Although emerging codes and themes were discussed within the research team to support interpretation, formal inter-coder agreement was not calculated. In addition, only 20 of 39 patients chose to involve a nominated social supporter, indicating that the social support component may not be acceptable or necessary for all patients. This reinforces the importance of keeping supporter involvement optional, consent-based, and patient-controlled.

Intervention-use records also have limitations. Message delivery records indicate that SMS reminders, educational messages, and supporter prompts were sent or recorded by the system, but delivery does not necessarily mean that messages were received, read, understood, or acted upon by participants. This distinction is especially important in a context where phone sharing, intermittent network access, charging problems, literacy barriers, and privacy concerns may affect message use. Finally, the study was conducted at a single referral hospital in southwestern Uganda, which may limit transferability to other settings. Further implementation studies are needed to assess longer-term integration, sustainability, objective adherence outcomes, clinical outcomes, and preliminary impact under routine care conditions.

## Conclusions

Participants generally perceived the intervention as acceptable and practical for supporting medication-taking and clinic care among adults living with HIV and type 2 diabetes in Uganda. Using a mixed methods and UCD approach, this study identified important insights on SMS reminders, educational messages, patient-controlled social supporter SMS prompts, discreet communication, clinic linkage, and the practical design considerations needed to support medication-taking and care coordination in a low-resource setting. The findings suggest that mHealth interventions for multimorbidity may be more acceptable when they strengthen optional patient-controlled social support, protect patient privacy, and fit within patients’ everyday treatment and communication practices. Further implementation research is needed to assess longer-term adoption, fidelity, sustainability, and preliminary impact under routine care conditions.
